# Paradoxical increase of neurofilaments in SMA patients treated with onasemnogene abeparvovec-xioi

**DOI:** 10.3389/fneur.2023.1269406

**Published:** 2023-12-13

**Authors:** Marina Flotats-Bastardas, Lisa Bitzan, Charlotte Grell, Kyriakos Martakis, Benedikt Winter, Michael Zemlin, Claudia D. Wurster, Zeljko Uzelac, Claudia Weiß, Andreas Hahn

**Affiliations:** ^1^Department of Pediatric Neurology, Saarland University Medical Center, Saarland University, Homburg, Germany; ^2^Department of Pediatric Neurology, Charité Universitätsmedizin Berlin, Berlin, Germany; ^3^Department of Child Neurology, Justus Liebig University Giessen, Giessen, Germany; ^4^Department of Pediatrics, Faculty of Medicine and University Hospital Cologne, Cologne, Germany; ^5^Department of Child Neurology, Mannheim University, Mannheim, Germany; ^6^Department of General Pediatrics and Neonatology, Saarland University Medical Center, Saarland University, Homburg, Germany; ^7^Department of Neurology, Ulm University, Ulm, Germany

**Keywords:** onasemnogene abeparvosec, SMA, neurofilament, nusinersen, risdiplam

## Abstract

**Background/Objective:**

Neurofilament light chain (NfL) has been proposed as a biomarker reflecting disease severity and therapy response in children with spinal muscular atrophy type 1 and 2 (SMA1 and 2). The objective of this study was to examine how serum NfL changes after gene replacement therapy (GRT) with onasemnogene abeparvovec-xioi.

**Methods:**

We measured NfL in serum probes from 19 patients (10 SMA 1 and 6 SMA 2; 15 previously treated with nusinersen or risdiplam; 12 male) before and at variable time points after GRT. These values were related to motor scores (CHOP-Intend, HFMSE and RULM).

**Results:**

Median age at GRT was 19 months (range 2–46 months). Median NfL of all patients before GRT was 39 pg/ml (range 0–663 pg/ml; normal values <25 pg/ml), increased significantly to 297 pg/ml (range 61–1,696 pg/ml; p<0,002) 1 month after GRT, and decreased to 49 pg/ml (range 24–151 pg/ml) after 6 months. Subjects pre-treated with nusinersen or risdiplam had lower baseline NfL levels than naïve patients (p<0,005), but absolute increases of NfL were similar in both groups. While motor scores were improved in 14 out of 18 SMA patients (78%) 6 months after GRT NfL values differed not significantly from those measured at baseline (p = 0,959).

**Conclusion:**

Serum NfL showed a paradoxical transient increase after GRT in both, pre-treated and naïve patients, which may reflect an immunological reaction in the CNS related to transfection of neuronal cells by AAV9. The clinical meaning of this increase should be assessed in future studies. Our findings encourage regular monitoring of NfL in OA treated patients.

## Introduction

Spinal muscular atrophy (SMA) is a neuromuscular disease caused by degeneration of the lower motor neuron leading to progressive muscle weakness and atrophy (1). The phenotype is broad and ranges from infants with rapidly progressive muscle weakness evolving within the first months of life (SMA1) to patients demonstrating first symptoms in adolescence or even adulthood (SMA3/4). While SMA is caused by biallelic mutations in the Survival Motor Neuron 1 (*SMN1*) gene, severity of disease is largely determined by the copy number of the paralogous gene *SMN2*. *SMN2* differs by 5 nucleotides from SMA1; which leads to alternative splicing reducing the amount of functional SMN protein to ~10% compared to *SMN1* (2).

In 2016, the antisense-oligonucleotide nusinersen was approved for treatment of SMA, followed by the gene replacement therapy (GRT) with onasemnogene abeparvovec-xioi (OA) in 2019, and the small-molecule risdiplam in 2020 (3–5). While nusinersen and risdiplam are splicing modifiers of the *SMN2* transcript OA releases an *SMN1* transgene. Although all three drugs have shown to substantially modify positively the course of disease in children with SMA by improving or stabilizing motor, respiratory, and bulbar function, biomarkers would be helpful for assessing disease severity as well as monitoring therapy response and guiding therapeutic decisions (6, 7).

Neurofilaments (Nfs) are neuronal cytoskeletal proteins that are believed to provide structural support for axons, to regulate axonal diameter, and to preserve structural axonal integrity. Nfs exist in a phosphorylated form with high molecular weight (NfH), a medium, and a light form (NfL) (6). It has shown that healthy children younger than age 4 years have higher serum NfL levels than older children and adolescents, which is thought to reflect physiological neuronal apoptosis during the first months of life (8, 9). In case of neuronal injury, increased Nfs can be measured in cerebrospinal fluid (CSF) and in peripheral blood, and are regarded as reliable biomarkers for several neurodegenerative diseases of the central (CNS) and peripheral nervous system (6, 7, 9). Higher levels of Nfs in plasma and CSF have also been found in infants and children with SMA1 and 2 compared to healthy ones. In addition, patients with 2 SMN2 copies had higher levels than those with >2 SMN2 copies (8–10). Studies in infants and younger children with SMA1 and 2 under therapy with nusinersen showed a decrease in Nfs during the first months of therapy as well as an improvement of motor function (8, 10, 14, 15). This suggests that Nfs could be used as biomarkers assessing severity of disease and response to therapy in infants and younger children with SMA1 and 2. Opposite to this, NF values in adolescents and adults with SMA2 and 3 differed not significantly from those in healthy controls indicating that they are less well suited in this age group (8, 11–13).

In contrast to nusinersen, there is hardly any NfL data available for SMA patients receiving OA (9). Therefore, the aims of our study were to investigate how NfL levels change after GRT, and whether they are suitable biomarkers reflecting therapy response in SMA patients treated this way.

## Methods

In Germany, patients with biallelic *SMN1* mutations and 2 or 3 *SMN2* copies are amenable to treatment with OA regardless of age, weight and pre-treatment status. By contrast, additional therapy with risdiplam or nusinersen after application of OA is usually not reimbursed by insurance companies.

In this retrospective multicenter study, we included 19 German SMA children treated with OA between March 1, 2020 and November 21, 2022 at the neuropediatric departments of the university hospitals Homburg (*n* = 4), Giessen (*n* = 3), Ulm (*n* = 1) and Berlin (*n* = 11), for whom at least one blood probe before and after OA treatment suitable for NfL determination had been stored or NfL determination had been performed as part of the clinical routine. All other OA treated patients were excluded from analysis.

All patients had a genetically confirmed 5 q-SMA (i.e., biallelic deletions and/or mutations in *SMN1*), and negative AAV9 titers (<1:50) determined within 4 weeks before GRT.

Patients underwent clinical examinations, blood analyses, and assessments of motor function at baseline as well as 1, 2 and 6 months after GRT, and thereafter every 4 months according to recommendations of the SMArtCARE consortium during their routine hospital stays (16, 17). Motor function was assessed by the Children's Hospital of Philadelphia Infant Test of Neuromuscular Disorders (CHOP-Intend), a validated rating scale for SMA1; as well as the Hammersmith Functional Motor Scale Expanded (HFMSE) and the Revised Upper Limb Module (RULM), both validated motor tests for SMA2 and 3 (18–20). The highest scores representing the best motor functions achievable are 64 points for the CHOP-Intend, 66 points for the HFMSE, and 37 for the RULM. Improvement of more than 4 points in CHOP-Intend score, 3 points in HFMSE and 2 points in RULM are thought to reflect clinical meaningful changes (3, 21, 22). Motor function tests were performed by experienced and trained physiotherapists.

The study was approved by the local ethic committees of the participating centers (Homburg: 288/20, Gießen: AZ134/18, Ulm: 19/12, Berlin: EA2/061/18). All legal guardians gave informed written consent to participate in the study.

### Sample analysis

NfL levels were determined in serum probes taken within 4 weeks before GRT (baseline) and 1, 2, 6, 10, and 14 months after GRT if available. In 13 participants (68%) more than 2 probes were available. Serum NfL concentrations in 8 patients (1 naïve, 7 pre-treatment) were measured at the Department of Neurology, Ulm University with the single molecular array (Simoa) platform provided by Quanterix (Lexington, MA, USA) (23, 24). In 11 patients (3 naïve, 8 pre-treated) NfL levels were determined at the Department of Pediatric Neurology, Charité Universitätsmedizin Berlin with the ELLA microfluid system (Bio-Techne, Minneapolis, USA) (25). The Simoa assay is considered to be the reference method, while the Ella assay allows a rapid and sensitive analysis. Results obtained by using the ELLA system highly correlate with those obtained with the Simoa essay (25).

Serum was obtained from peripheral blood by centrifugation (800 g, 5 min) and was stored within 2 h at −80°C if probes were not analyzed on the same day. As previously reported, serum NfL levels < 25 pg/mL (upper range of normal values) were considered normal regardless of the technique applied (8, 9).

### Statistical analysis

Since many variables were not normally distributed, continuous variables were expressed as median values and range. The Mann-Whitney test was used to compare variables between 2 groups and the Wilcoxon signed rank test was applied to analyze differences of variables between more than 2 groups. No adjustment for multiple testing was done. Correlations of serum NfL levels with motor scores were analyzed with Spearman Rho. A two-sided p value ≤ 0.05 was interpreted as statistically significant. Statistical analysis was performed with IBM^Ⓡ^ SPSS^Ⓡ^ Statistics.

## Results

### Patient characteristics

Patient demographics are summarized in Table 1 and data for each single patient are shown in Supplementary Table 1. 10 patients were diagnosed as SMA 1, 6 as SMA2, and 1 as SMA3. 11 patients had 2, 7 had 3 *SMN2* copies, and 1 had 4 *SMN2* copies. Two patients were pre-symptomatic. One patient had 3 *SMN2* copies and one had 2 *SMN2* copies. Median age at GRT was 19 months (range 2–46 months) with a median weight of 9,8 kg (range 6–15 kg). GRT was performed as first line therapy in 8 patients (42%). Reasons for GRT in the remaining 11 subjects (58%) were inadequate motor progress or motor regression in 5 (26%), respiratory deterioration in 1 (5%), and minor burden compared to repetitive lumbar punctures in 5 (26%). Before GRT, 4 patients (21%) were ventilated invasively or non-invasively. 3 patients (16%) were able to sit without support, 3 (16%) were able to stand or walk with support, and 1 (2%) walked without support. During follow-up one patient died at age 5 months because of aspiration pneumonia. All other patients were alive at last follow-up. The last nusinersen dose was applied 4 to 21 weeks (median 12 weeks) before GRT.

**Table 1:** Patient demographics and Neurofilament light chain (NfL) serum values before gene replacement therapy (GRT) for 19 patients with SMA.

**Patient demographics**							
	**Pre-treated**	**Naive**	≤ **2 SMN2**	>**2 SMN2**	**NfL** ≤ **25pg/mL**	**NfL** > **25 pg/mL**	**Total**
*n* (%)	15 (79)	4 (21)	11 (58)	8 (42)	8 (42)	11 (58)	19 (100)
Median age (range), months	18 (2–46)	15,5 (7–18)	11 (2–46)	21,5 (5–44)	27,5 (4–46)	12 (2–31)	19 (2–46)
Sex *n* (%), male	10 (83)	2 (17)	7 (58)	5 (42)	6 (50)	6 (50)	12 (63)
SMA type 1, *n* (%)	9 (90))	1 (10)	10 (100)	0	2 (20)	8 (80)	10 (53)
SMA type 2, *n* (%)	3 (50)	3 (50)	0	6 (100)	3 (50)	3 (50)	6 (32)
SMA type 3, *n* (%)	1 (100)	0	0	1 (100)	1 (100)	0	1 (5)
2 SMN2 copies, *n* (%)	10 (91)	1 (9)	11 (100)	0	3 (27)	9 (73)	11 (58)
Nusinersen	13 (100)	0	10 (77)	3 (23)	6 (46)	7 (54)	13 (68)
Risdiplam	2 (100)	0	0	2 (100)	2 (100)	0	2 (11)
Median weight at GRT (range)	9 (6–15)	9,4 (8–11)	9 (6–15)	9,4 (8–11)	12,1 (6–15)	8,3 (6–14)	9,8 (6–15)
G-Tube: *n* (%)	4 (100)	0	4 (100)	0	2 (50)	2 (50)	4 (21)
Assisted ventilation *n* (%)	4 (50)	4 (50)	4 (100)	0	2 (50)	2 (50)	4 (21)

### Serum Nfl values

**Figure 1:**
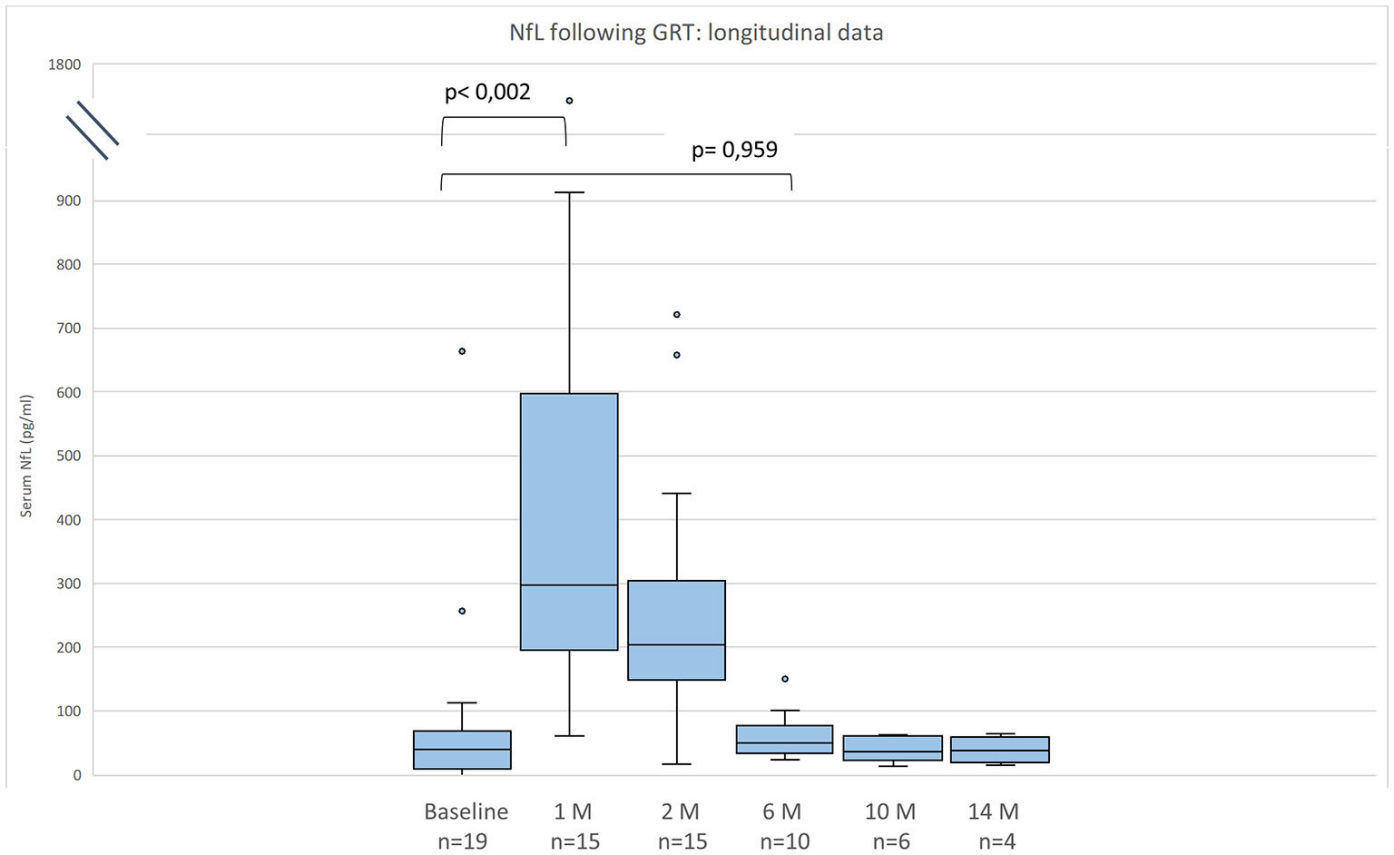
Neurofilament light chain (NfL) serum levels for 19 patients with SMA before and after gene replacement therapy (GRT). Given are median, minimum, maximum, and interquartile range.

**Figure 2:**
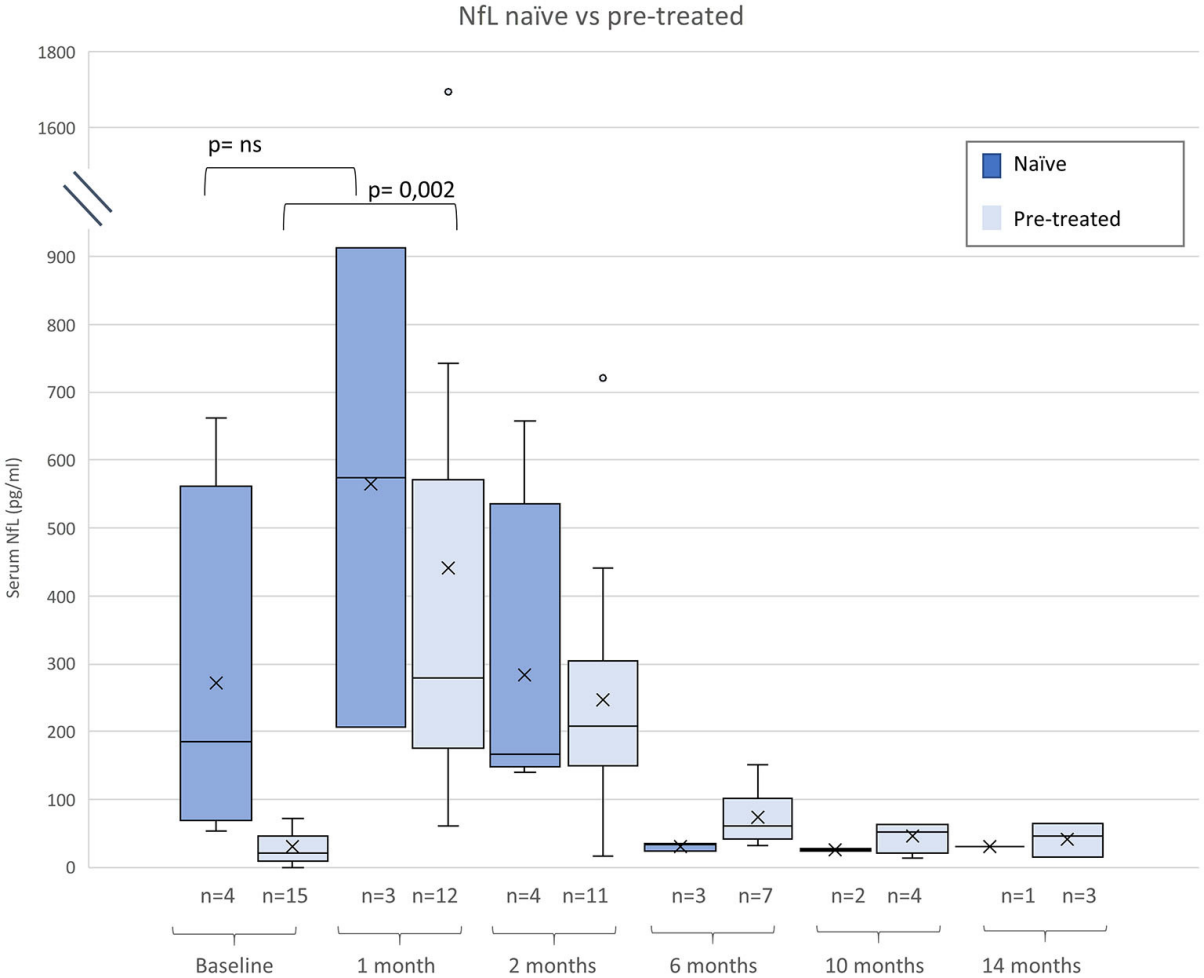
Neurofilament light chain (NfL) serum levels before and after gene therapy (GRT) for naïve and patients pre-treated with nusinersen or risdiplam. Given are median, minimum, maximum, and interquartile range.

NfL levels are shown in Table 2 and in Figures 1, 2. Individual data are presented in Supplementary Table 2. Mean follow-up time was 8 months (range 1 - 20 months). Across the study, there were no statistical differences between NfL concentrations and employed assay. NfL values at baseline were normal in 8 patients (42%), mildly increased (26–100 pg/ml) in 8 (42%), and distinctly elevated (>100 pg/ml) in 3 (16%). In the pre-treated group, 8 patients (53%) had normal NfL values at baseline and 7 (47%) had mildly increased (26–100 pg/ml) values.

**Table 2:** Neurofilament light chain (NfL) serum levels for 19 patients with SMA before and after gene replacement therapy (GRT).

**Neurofilament light chain serum levels before and after GRT**						
	**Before**	**1 month**	**2 months**	**6 months**	**10 months**	**14 months**
**All patients**, ***n***	19	15	15	10	6	4
Median NfL (range): pg/ml	39 (0–663)	297 (61–1696)	204 (16–721)	49 (24–151)	37 (13–63)	38 (15–64)
**Naiv**, ***n***	4	3	4	3	2	1
Median NfL (range): pg/ml	185 (55–663)	574 (206–913)	167 (141–658)	34,3 (24–35)	26,5 (25–28)	31 (–)
**Pre–treated**, ***n***	15	12	11	7	4	3
Median NfL (range): pg/ml	22 (0–73)	279 (61–1696)	208 (16–721	62 (32–151)	52,5 (13–63)	46 (15–64)
**≤2 SMN2**, ***n***	11	8	8	5	3	2
Median NfL (range): pg/ml	45 (7–663)	246 (61–743)	204 (16–721)	57 (32–151)	60 (13–63)	55 (46–64)
**>** **2 SMN2**, ***n***	8	7	7	5	3	2
Median NfL (range): pg/ml	10 (0–256)	489 (206–1696)	204 (141–441)	34,8 (24–69)	28,1 (25–45)	22,9 (15–31)
**NfL** **>** **25 pg/mL**, ***n***	11	8	9	8	3	2
Median NfL (range): pg/ml	55 (27–663)	374 (83–913)	168 (16–658)	37,9 (24–151)	28,1 (25–60)	38,4 (31–46)
**NfL** **≤25 pg/mL**, ***n***	8	7	6	2	3	2
Median NfL (range): pg/ml	8,5 (0–22)	260 (61–1696)	256 (149–721)	65,6 (62–69)	45 (13–63)	39,5 (15–64)
**Simoa (** ***n*** **)**	8	4	6	4	4	3
Median NfL (range): pg/ml	29 (0–663)	671 (170–1696)	323 (149–721)	59,5 (41–102)	52,5 (13–63)	46 (15–64)
**Ella (** ***n*** **)**	11	11	9	6	2	1
Median NfL (range): pg/ml	45 (5–256)	260 (61–913)	168 (16–304)	34,6 (24–151)	26,5 (25–28)	31 (–)

Compared to baseline NfL values were increased 1 month after GRT (p < 0,002, n = 15), and had returned to baseline 6 months after GRT (p = 0,959, n = 10) (Figure 1). NfL values at last follow-up were normal in 2 patients (11%), mildly increased (26–100 pg/ml) in 8 (42%), and distinctly elevated (>100 pg/ml) in 8 (42%).

NfL values at last follow-up were lower than at baseline in 7 patients (47%) and higher in 8 patients (53%).

Compared to naïve patients pre-treated children had lower baseline NfL levels (p < 0.005), Subjects pre-treated with nusinersen or risdiplam had lower baseline NfL levels than naïve patients (p < 0.005), but absolute increases of NfL were similar in both groups (Figure 2).

### Motor function 6 months after GRT

Motor function was measured at baseline and 6 months after GRT in 18 patients (95%). Overall, motor function improved in 14 out of 18 (78%) patients, deteriorated in 2 (11%), and remained unchanged in 2 (11%). Clinically meaningful changes in at least one the motor tests applied was observed in 11 (61%) patients (CHOP-Intend score 7/8 = 88%), (HFMS 4/4 = 100%), and RULM (1/3 = 33%). Compared to motor status at baseline 14/19 (74%) patients acquired a new motor milestone: 4 instead of 3 (+1) were able to stand or walk with support and 6 instead of 1 (+5) walked without support.

When relating NfL concentrations to motor status values declined in 7 out of 14 patients (50%, 4 SMA1 and 3 SMA2) who improved in motor function testing and increased in 2 out of 2 patients (100%, 1 SMA1, 1 SMA3) who deteriorated in motor function. Motor scores in relation to NfL levels at baseline and 6 months after GRT as well as the changes of NfL values and motor scores showed no significant correlations and are depicted in Supplementary Figures 1, 2.

## Discussion

The aims of this study were to assess how NfL values change after GRT in SMA, and whether NfL is a suitable biomarker for assessing therapy response in SMA1 and 2 patients receiving OA. Our main finding was that NfL paradoxically increased significantly within the first month after GRT and returned to baseline values within 6 months; eventually reflecting an inflammatory response in the central nervous system due to AAV9 transfection of neuronal cells. This paradox occurred in naïve subjects as well as in individuals pre-treated with nusinersen or risdiplam. These findings suggest that regular monitoring of NfL within the first months after treatment with OA is meaningful.

Elevated NfL and NfH values have been demonstrated in different neurodegenerative and neuromuscular disorders such as Alzheimer's disease, amyotrophic lateral sclerosis, and Charcot-Marie-Tooth disease (6). Increased Nf values have also been reported for treated and untreated SMA1 and 2 patients; with untreated SMA1 patients and/or patients with 2 *SMN2* copies having higher values than those with SMA2 and/or 3 *SMN2* copies. In the ENDEAR study assessing the effects of nusinersen in symptomatic SMA1 patients, plasma NfH values declined in both, treated and untreated SMA1 patients, over time, but this decrease occurred much more rapidly in patients receiving nusinersen. However, NfH values remained increased in at least some patients despite treatment (10). In the NURTURE trial evaluating the outcome of pre-symptomatic infants with SMA, plasma NfH values measured prior to treatment and on day 64 were the strongest predictors of future milestone achievement (26). Collectively, our finding of highly variably increased NfL values at baseline in a heterogeneous cohort with regard to age and treatment status is in line with previous studies in SMA1 and patients with 2 and 3 *SMN2* copies (8, 10–13, 15, 27).

Studies analyzing NfL in patients undergoing GRT are scarce. Only Alves et al. analyzed NfL approximately every 100 days following treatment with OA in 7 naïve and 6 SMA patients pre-treated with nusinersen (9). Similar to what was observed in our study they found that serum NfL raised initially and decreased over time in almost all patients, even though all experienced motor improvement. Similarly to our findings, they observed that NfL did not increase in 3 patients pre-treated with nusinersen within 2 weeks prior to GRT, and that the increase was less prominent than in drug-naïve patients when the last nusinersen dose was applied more than 3 weeks before GRT. Based on these findings they speculated that pre-treatment with nusinersen could have a protective effect on the concomitant inflammatory response related to GRT.

In our study we observed an increase of NfL within the first 30 days post GRT in all 15 patients (100%) for whom serum probes before and 1 month after GRT were available. This incline was highly variable. Median NfL values at baseline and at one month post GRT were significantly lower in pre-treated subjects than in naïve individuals, but a distinct increase after GRT occurred in both groups (from 22 to 279 pg/ml in pre-treated and from 185 to 574 pg/ml in naïve patients). Therefore, our findings do not support the notion that a preceding nusinersen or risdiplam therapy reduces a potential intrathecal immune response. However, lower NfL values at baseline in pre-treated patients can be interpreted as reduced neuronal death, demonstrating the principal value of a bridge therapy until OA is effective (9).

Several studies on nusinersen treated patients with SMA1 and 2 have shown a reduction of NfH and NfL levels in cerebrospinal fluid (CSF) and blood with concomitant improvement in motor scores (8–10, 15). But, as in our cohort, NfL levels may remain ~3-fold higher in some individuals than in aged-matched controls (8). In the present study we documented an improvement of motor function in 78% and a clinically meaningful amelioration in about two thirds of patients during the analysis period, which is in congruence with previous investigations analyzing efficacy of nusinersen and GRT (3). However, in divergence to others, we could not find a correlation between NfL levels and motor scores. This discrepancy could be explained by the heterogeneous cohort with regard to age and pre-treatment investigated in the present study.

Clinical trials and real-world studies have documented a multisystemic inflammatory response against AAV9 within the first weeks after GRT (4, 28). Fever, vomiting, and laboratory signs of liver inflammation are frequent side effects related to GRT, while acute liver failure and multisystemic thrombotic microangiopathy represent rare severe complications. A prednisolone therapy for almost 2 months after GRT is recommended in order to reduce the inflammatory response and to avoid severe side effects (29). A specific immunologic process in the CNS in humans has not yet been documented and is not listed as a potential side effect in the package leaflet. However, such a local immunological reaction would not be completely unexpected due to the CNS trophism of the AAV9 vector used for gene delivery. This hypothesis is also supported by a study in non-human primates documenting minimal neuronal degeneration and cell inflammation in dorsal root ganglia and trigeminal ganglia, as well as minimal axon degeneration in the spinal cord 6 weeks after GRT (30).

This retrospective study has several shortcomings. The number of patients with complete data sets was relatively small, making reliable subgroup analyses difficult. Moreover, the study cohort was heterogeneous with respect to age and pre-treatment status. In addition, NfL were determined with 2 different techniques, making an ascertainment bias possible, although a strong correlation was found between both platforms (25). Finally, no other parameters supporting an inflammatory intrathecal reaction (e.g. CSF cell count, protein, oligoclonal bands) have been analyzed.

However, our results show a transient increase of NfL values in close temporal relation to GRT, pointing to a potential CNS inflammatory response against the AAV9 vector. Future prospective studies including a larger cohort of patients and using the same assay, preferably the Simoa platform, have to clarify whether this interpretation is correct. Our findings encourage regular monitoring of NfL in OA treated patients.

## Data availability statement

The original contributions presented in the study are included in the article/Supplementary material, further inquiries can be directed to the corresponding author.

## Ethics statement

The studies involving humans were approved by the local ethic committees of the participating centers (Homburg: 288/20, Gießen: AZ134/18, Ulm: 19/12, Berlin: EA2/061/18). The studies were conducted in accordance with the local legislation and institutional requirements. Written informed consent for participation in this study was provided by the participants' legal guardians/next of kin. Written informed consent was obtained from the minor(s)' legal guardian/next of kin for the publication of any potentially identifiable images or data included in this article.

## Author contributions

MF-B: Conceptualization, Data curation, Formal analysis, Writing—original draft. LB: Data curation, Writing—review & editing. CG: Data curation, Writing—review & editing. KM: Writing—review & editing. BW: Data curation, Writing—review & editing. MZ: Writing—review & editing. CDW: Investigation, Writing—review & editing. ZU: Formal analysis, Investigation, Writing—review & editing. AH: Methodology, Supervision, Writing—original draft. CW: Investigation, Writing—review & editing.
