# Supplementary material for: Paradoxical increase of neurofilaments in SMA patients treated with onasemnogene abeparvovec-xioi
Source: Front Neurol. 2023 Dec 13;14:1269406. doi: 10.3389/fneur.2023.1269406 (PMC10756901; doi:10.3389/fneur.2023.1269406)

Supplementary data

Table S1: Patient demographics and motor function scores before and after gene replacement therapy (GRT). SMA: spinal muscular atrophy. SMN: survival motor neuron. GRT: gene replacement therapy. CHOP: Children's Hospital of Philadelphia Infant Test of Neuromuscular Disorders, HFMSE: Hammersmith Functional Motor Scale Expanded. RULM: Revised Upper Limb Module. Pre: presymptomatic

Table S2: Individual data serum NfL levels after and following GRT.

Supplementary figure 1: Correlations between NfL values and motor scores before and after 6 months GRT. CHOP: Children's Hospital of Philadelphia Infant Test of Neuromuscular Disorders, HFMSE: Hammersmith Functional Motor Scale Expanded, RULM: Revised Upper Limb Module (RULM)


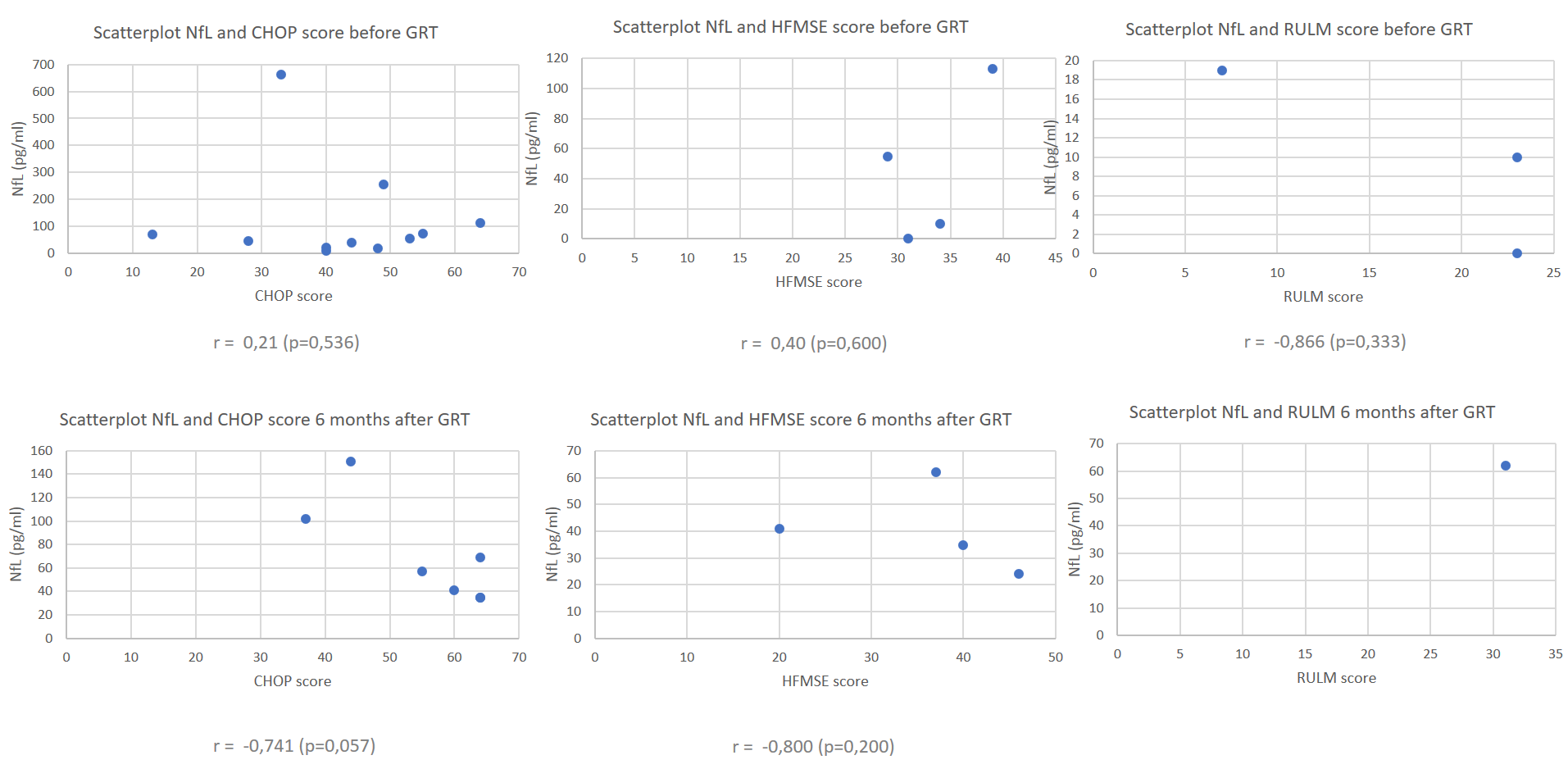


Supplementary figure 2: Correlations between absolute difference between baseline und last measurement NfL values with absolute difference motor scores scale between baseline and last measurement. CHOP: Children's Hospital of Philadelphia Infant Test of Neuromuscular Disorders, HFMSE: Hammersmith Functional Motor Scale Expanded, RULM: Revised Upper Limb Module (RULM)


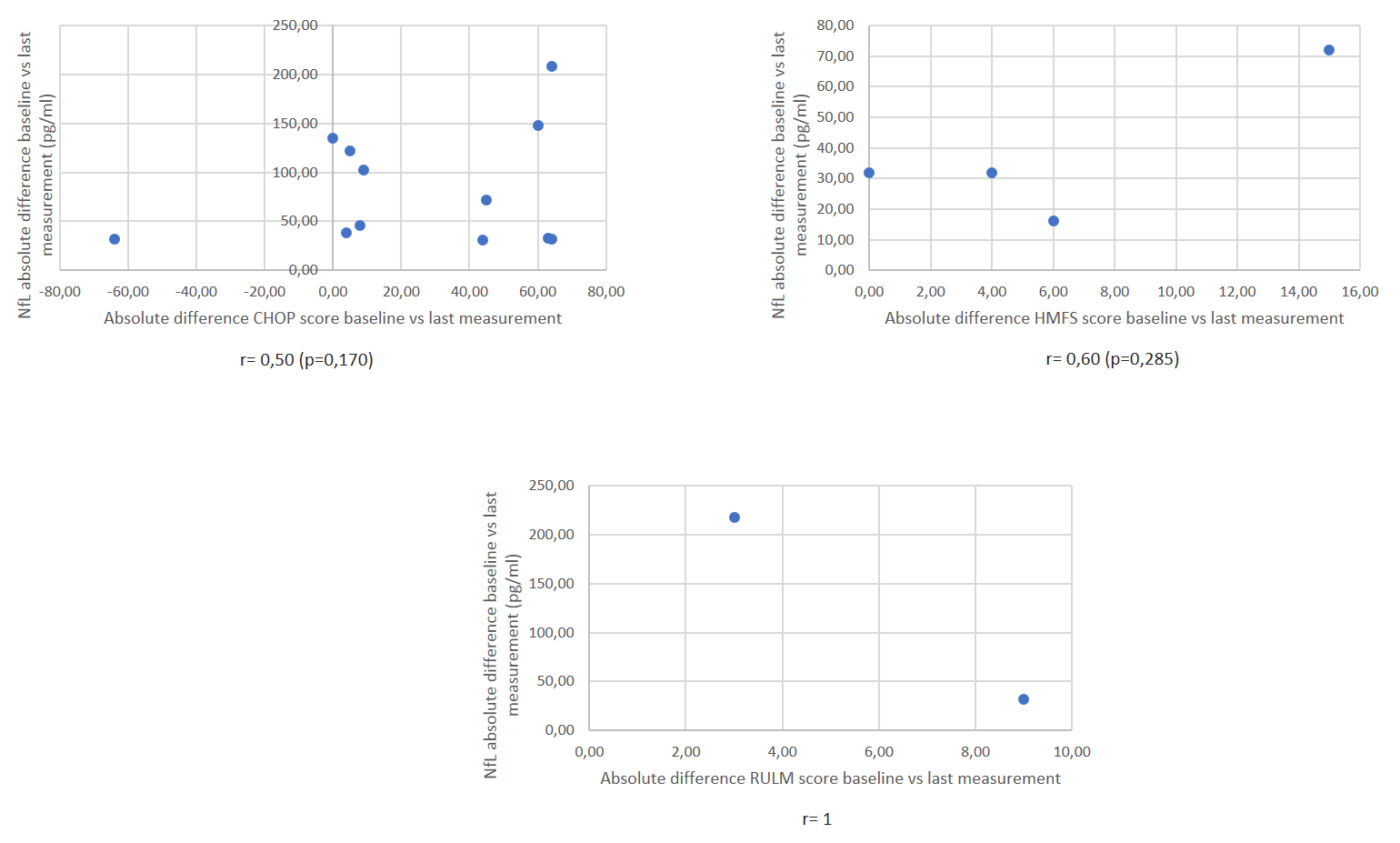

Supplement: Supplementary file 1 [file Data_Sheet_1.docx]
